# Tobacco cessation Clinical Practice Guideline use by rural and urban hospital nurses: a pre-implementation needs assessment

**DOI:** 10.1186/1472-6955-11-6

**Published:** 2012-04-30

**Authors:** Patricia M Smith, Scott M Sellick, Michelle M Spadoni

**Affiliations:** 1 Division of Human Sciences, Northern Ontario School of Medicine, 955 Oliver Rd, Thunder Bay, ON P7B 5E1, Canada; 2 Thunder Bay Regional Health Sciences Centre, 980 Oliver Road, Thunder Bay, ON P7B 6 V4, Canada; 3 School of Nursing, Lakehead University, 955 Oliver Rd, Thunder Bay, ON P7B 5E1, Canada

## Abstract

**Background:**

This study was a pre-program evaluation of hospital-based nurses' tobacco intervention beliefs, confidence, training, practice, and perceived intervention barriers and facilitators. It was designed to identify relevant information prior to implementing tobacco cessation guidelines across a large northern rural region, home to 1 urban and 12 rural hospitals.

**Methods:**

This cross-sectional survey was distributed by nurse managers to nurses in the 13 hospitals and returned by nurses (N = 269) via mail to the researchers.

**Results:**

Nurses were somewhat confident providing cessation interventions, agreed they should educate patients about tobacco, and 94% perceived tobacco counselling as part of their role. Although only 11% had received cessation training, the majority reported intervening, even if seldom--91% asked about tobacco-use, 96% advised quitting, 89% assessed readiness to quit, 88% assisted with quitting, and 61% arranged post-discharge follow-up. Few performed any of these steps frequently, and among those who intervened, the majority spent < 10 minutes. The most frequently performed activities tended to take the least amount of time, while the more complex activities (e.g., teaching coping skills and pharmacotherapy education) were seldom performed. Patient-related factors (quitting benefits and motivation) encouraged nurses to intervene and work-related factors discouraged them (time and workloads). There were significant rural-urban differences--more rural nurses perceived intervening as part of their role, reported having more systems in place to support cessation, reported higher confidence for intervening, and more frequently assisted patients with quitting and arranged follow-up.

**Conclusions:**

The findings showed nurses' willingness to engage in tobacco interventions. What the majority were doing maps onto the recommended minimum of 1-3 minutes but intervention frequency and follow-up were suboptimal. The rural-urban differences suggest a need for more research to explore the strengths of rural practice which could potentially inform approaches to smoking cessation in urban hospitals.

## Background

Although there have been great achievements in the decline of smoking over the last few decades and concomitant decreases in smoking-related diseases and mortality [1], smoking cessation has slowed in Canada [2] and smoking rates in rural areas remain markedly higher than in urban areas [3]. Integrating cessation interventions into daily nursing practice and offering assistance to all patients who smoke is one strategy to increase smoking cessation [4] since few people seek out cessation assistance on their own [5]. This can be especially important in acute care settings where patients tend to be receptive and cessation interventions have been found to be exceptionally effective [6,7]. The aim of the present study was a pre-program evaluation of hospital-based nurses' tobacco intervention beliefs, confidence, training, practice, and perceived intervention barriers and facilitators. It was designed to identify relevant information prior to implementing tobacco cessation guidelines across a large northern rural region, home to 1 urban and 12 rural hospitals.

In Canada, professional nursing associations, such as the Registered Nurses Association of Ontario (RNAO) [4], have developed Best Practice Guidelines for tobacco interventions consistent with, and based on, the Public Health Service (PHS) Tobacco Use and Dependence Clinical Practice Guideline [8]. The PHS Guideline is the most extensive published guideline because it includes myriad meta-analyses on which the Guideline was based. First published in 1996 by the Agency for Healthcare Policy and Research (AHCPR) [9] (now the Agency for Healthcare Research and Quality [AHRQ]), and updated in 2000 [10] and 2008 [8], the Guideline recommends that all clinicians intervene for a minimum of 1-3 minutes with all patients on every encounter using the 5A protocol--*ask*, *advise*, *assess*, *assist*, and *arrange *follow-up. It also recommends five systems-level strategies that have been found to significantly increase the delivery of treatments [8]: 1) systems for the identification and documentation of tobacco use; 2) provider training, patient resources, and provider feedback; 3) designated staff to provide interventions; 4) policies to support cessation; and 5) provision of cessation counselling and pharmacotherapy at no cost to patients. In earlier versions of the Guideline, a sixth strategy was identified for appropriate recognition/reimbursement for interventions [10], which has now been subsumed under the 4^th ^recommendation (policies) [8].

Although little information is available about nurses' tobacco-related beliefs and 5A practice in rural settings, there is a growing literature on what nurses are doing in relation to the 5A protocol in urban hospitals. One of the largest and more recent studies to report on nurses' adherence to the 5A protocol involved 35 hospitals in the United States (USA); the findings showed moderately good adherence to frequently initiating the 5A protocol (73% ask, 62% advise, and 62% assess) but relatively poor adherence for assisting (37%) and arranging (22%) [11]. Similar findings have been reported in moderate-sized urban hospitals in the Canadian province of Manitoba, with 68-81% of nurses reporting almost always or frequently asking, 32-46% advising, 51-57% assessing, 21-35% assisting, and 3-38% arranging [12]. Likewise, internationally, such as in Hong Kong and other cities in China, nurses are more likely to initiate (ask, advise, and assess), and less likely to assist and arrange follow-up [13,14].

Overall, most studies show that adherence to the 5A protocol is encouraging and has been increasing over the last decade, but it still tends to be suboptimal with nurses reporting that they feel unprepared and have limited institutional support for assisting with cessation [12]. Nurses who smoke are less likely to intervene [11], and other common barriers to intervention include the perceived lack of necessary skills, time, and patient motivation, and perceptions that providing advice or brief interventions is ineffective [15]. For the current study, it was not clear how receptive nurses would be to following the 5A protocol because in addition to possibly encountering the barriers identified in the literature, smoking tends to be part of the social fabric of the rural communities that participated in this study, especially for younger adults. The overall smoking prevalence in the rural communities was 32% compared to 22% in the urban centre, with rural rates ranging as high as 41-47% for 20-44 year-olds [16]. In an Australian study, where, similar to Canada, rural smoking rates are high and have been symbolically associated with rural values such as self-reliance, hardiness, and friendship, 53% of rural nurses were found to advise patients to quit smoking, which is lower than most other studies [11-14]; unfortunately, the authors did not report on the other 5A steps [17].

The current study was part of a pre-program evaluation of a large 4-year project designed to increase the capacity of northern hospitals to implement and deliver smoking cessation interventions. The specific questions for the pre-program evaluation were: 1) To what extent do nurses see providing tobacco interventions as part of their role? 2) How confident are nurses in providing tobacco interventions consistent with the 5A protocol? 3) To what extent and with what frequency do nurses adhere to the 5A protocol in practice and what types of treatments are they providing? 4) What do nurses perceive as barriers and facilitators to intervening? 5) What proportion of nurses has received training and what type of training would they prefer? and, 6) What systems strategies are in place in the hospitals? We also examined the similarities and differences of the nurses who worked in the rural hospitals compared to those who worked in the larger urban hospital. These comparisons were made because data are lacking for nurses working in rural hospitals, and to avoid overweighting the outcomes by the one urban hospital which had more than two times the number of nurses employed relative to all the rural hospitals combined.

## Methods

### Design and participants

The study involved a cross-sectional survey with hospital-based registered nurses in acute care hospitals in NW Ontario which were the intended targets for the implementation of tobacco cessation Clinical Practice Guidelines [8] into daily nursing practice. The study received ethics clearance through the regional hospital (Thunder Bay Regional Health Sciences Centre) institutional review board (IRB), which also served as the IRB for 11 of the hospitals (TBRHSC REB #42-06); two of the community hospitals (Sioux Lookout Meno-Ya-Win Health Centre, and Lake of the Woods District Hospital) provided additional clearance from their own IRBs.

### Setting

Hospitals in Canada are publicly funded under a provincially-run single-tier universal healthcare system. The catchment areas for hospitals in the province where the study took place (Ontario) are defined geographically by the Local Health Integration Network (LHIN) regionalization structure. NW Ontario is LHIN 14; it covers a large land mass (526,355 square kilometres--almost 50% of the province) but is sparsely populated (approximately 235,000 or 2% of the provincial population) [18]. All hospitals in LHIN 14 agreed to participate. One was the regional tertiary hospital located in the only urban centre in NW Ontario (Thunder Bay, population 109,145); the other 12 hospitals were in rural communities, with populations ranging from 910-8,190 for 11 of the communities--Atikokan, Dryden, Fort Frances, Geraldton, Hornepayne, Marathon, Manitouwadge, Nipigon, Red Lake, Sioux Lookout, and Terrace Bay--and 15,180 for one community--Kenora [19].

### Procedure

Nurses in all 13 hospitals were invited to attend information sessions where the purpose of the survey was explained by the researchers. Study posters were placed around the hospitals encouraging participation. The researchers provided hospitals with the surveys, information letters, and informed consent forms which were distributed to nursing staff by their managers primarily during departmental nursing meetings; one hospital attached the surveys to pay-stubs. Completion of the surveys was voluntary. All surveys included a stamped return envelope addressed to the investigators and hospitals were provided with a tracking sheet to record the number of surveys distributed.

### Survey instrument

#### Attitudes

Four items from a published scale [13] measuring attitudes about providing tobacco interventions were used. The question stem was: "In relation to your work with hospitalized patients, please indicate how strongly you agree or disagree with the following statements" (1 = strongly disagree, 2 = disagree, 3 = agree, and 4 = strongly agree). The items were: "Health education on the risk of smoking is an important part of nursing care"; "Nurses should use every opportunity to educate clients on the health effects of smoking"; "Nurses should educate other smokers in the clients' household if at all possible"; and, "Nurses should advise clients to quit smoking even if help is not requested". Scale reliability was high; α = 0.82 in the original study [13] and α = 0.79 in the current.

#### Beliefs

A single item measuring whether brief advice to help clients stop smoking was effective [13] was measured using a scale of strongly disagree (1) to strongly agree (4).

#### Confidence

Eight items assessing nurses' perceived confidence to provide tobacco interventions were measured on a 4-point scale (1 = not at all confident, 2 = somewhat confident, 3 = confident, and 4 = very confident). The question stem was: "In relation to your work with hospitalized patients, please indicate how confident you feel or would feel performing the following tasks"; the items were: "Teaching smokers about the general health risk of smoking"; "Finding out about smokers' beliefs about smoking and health"; "Counteracting smokers' negative attitudes about giving up smoking"; "Advising smokers on how to quit smoking"; "Negotiating a target date for clients to quit smoking"; "Discussing different methods of quitting smoking"; "Giving advice about nicotine replacement therapy"; and, "Using leaflets and other written materials to help clients quit smoking". The wording was modified slightly from a published scale [13], replacing "competence" with "confidence" because the relationship between confidence [self-efficacy] and behaviour is better-established [20]. Scale reliability was high; α = 0.90 for the original study [13] and α = 0.91 for the current.

#### Interventions in practice

Eighteen items assessing what types of cessation interventions nurses were providing in practice were measured on a 4-point scale (1 = never, 2 = seldom, 3 = occasionally, and 4 = frequently) with results presented as a mean score for each item. The question stem was: "In relation to your work with hospitalized patients, please indicate how often you performed the following activities in the past 12 months" followed by the list of items. Fourteen items were from a published scale [13], and four new items were developed: 1) recommend or suggest NRT; 2) recommend or suggest bupropion; 3) instruct on the use of pharmacotherapy; and, 4) help patients to set a quit date. A measure of the global 5A protocol steps (*ask, advise, assess, assist*, and *arrange*) was achieved by grouping the 18 practice items into the appropriate 5A steps. Scale reliabilities were acceptable: advise α = 0.83, assess α = 0.73, assist α = 0.90, and arrange α = 0.75.

#### Facilitators and inhibitors to practice

Thirteen factors that facilitate and eighteen inhibitors to providing tobacco cessation interventions were used, based on a published scale [13] using a "check all that apply" type list. The question stem was "Which of the following items discourage (encourage) you from advising or counselling patients to quit using tobacco?" followed by the list of discouraging/encouraging factors. Six of the 18 inhibitors included were new items developed for this study based on the Guideline systems-level recommendations [8]: limited direct patient care, no existing hospital mandate or policy to intervene, lack of patient interest or motivation to quit, lack of availability of educational materials, belief that unwanted advice upsets the provider-patient relationship, and limited effectiveness of tobacco cessation interventions. An open-ended comment field was provided for inhibiting and facilitating factors.

#### Cessation training

The preferred method for future training and training topics of interest were measured using a "check all that apply" type list designed for this study based on tobacco activities suggested in the Guideline [8]. The question stem was: "Which of the following resources would you use to learn more about tobacco cessation for patients? (Please check all that apply)--brief in-service (e.g., 10-minutes) during departmental meetings, 1-hour workshop, 1/2 day workshop, full-day workshop, and self-study materials (e.g., video/CD/DVD, books, pamphlets, web, etc.)." The stem for training topic items was: "If you were to receive tobacco cessation training, or further training, what areas would you like to know more about?" (Please check all that apply)"--how to: ask patients about tobacco use, advise patients to quit, assess readiness to quit, assist with quitting (provide social support, recruit social support, counsel, what self-help materials to provide, what to do if a patient continues to smoke), arrange follow-up (find/recommend post-discharge services), and how to organize your office in terms of record keeping and patient flow so that tobacco use status of patients is assessed at follow-up visits.

#### Systems

Items to measure the systems-level Guidelines [8] were developed for this study: "Does the hospital have a written protocol/policy for identifying tobacco use and tobacco cessation counselling?" (yes/no/unsure); "Are any of the following materials available in the hospital's waiting rooms, lounges, or patient rooms?" (posters encouraging cessation, pamphlets or self-help materials on tobacco cessation, quit line contact information, community-based tobacco cessation program information [yes/no/unsure]); "To what extent do you feel that delivering a tobacco cessation intervention is a part of your role as a healthcare provider?" (very much, somewhat, not at all); and, "Have you received any tobacco cessation training?" (yes/no).

### Data analyses

Means and standard deviations were used for beliefs, confidence, and practice items; frequency counts and percentages were used for inhibitors, facilitators, and systems outcomes. The global 5A steps were computed using means and standard deviations and also calculated as percentages of "ever intervene" to provide a measure of general openness to performing the steps regardless of the frequency with which nurses intervened ("*ask, advise, assess, assist*, and *arrange*" were coded as "yes" if respondents *ever *performed any of the activities for a given step), and "frequently intervene" to provide a measure of the frequency of adherence to the Guidelines [8] (5A steps were coded as "yes" if respondents performed any of the activities for a given step frequently). Inhibiting and facilitating factors were reported by categorizing them into knowledge/skills/attitudes (i.e., personal factors) and organizational (i.e., workplace factors) for ease of understanding. To test for rural and urban differences, chi-square was used for categorical and dichotomous comparisons and t-tests for continuous variable comparisons. To maintain the probability of "family-wise" Type 1 errors occurring at α = 0.05, a Bonferroni adjustment for the family-wise comparisons was used (n tests/.05), which resulted in the p value being set at α = .01 for the family comparisons of global 5A's, demographics, beliefs/confidence/time, systems, and intervention activities for "advise" and "assist", and at α = .004 for facilitating factors and α = .003 for inhibiting factor comparisons. For "ask", "arrange", and "assess" activity comparisons, the p value was set at α = .05, α = .03, and α = .02, respectively.

## Results

### Response rate

A total of 269 nurses from 12 of the 13 participating hospitals returned completed surveys--116 from rural hospitals, 142 from the urban hospital, and 11 that did not indicate a hospital. Written informed consent was received for all completed surveys. The nursing managers did not track the total number of surveys they distributed or return the unused surveys, so a true response rate cannot be calculated. However, using the total number of nurses employed at the hospitals at the time of survey distribution (N = 1627), a conservative estimate of the overall response rate, excluding the 11 respondents without a hospital indicated, is 13% for the urban hospital (142/1055) and 20% for the rural hospitals (116/572; range 10-45%). For the analyses, 257 of the 269 surveys received were used; excluded were the 11 surveys without a hospital identified (which was needed for the urban/rural comparisons) and 1 survey from the urban hospital because the respondent did not have direct patient contact.

### Participants

Participant characteristics are in Table 1. The majority of respondents worked full-time, had graduated on average in the mid to late 1980's, and had worked 13-14 years in their current area of practice. Most respondents were former or never smokers, and among smokers, most were either quitting or contemplating quitting. Although respondents held positive attitudes about intervening, few agreed that brief advice to quit smoking was effective. Most reported spending time counselling on tobacco, with the majority spending less than 10 minutes. Rural nurses were significantly more likely to perceive tobacco cessation counselling to be "very much" part of their role and had significantly higher counselling confidence (Table 1).

**Table 1:** Description of sample population

	Rural (N = 116)	Urban (N = 141)	p value
Current work status full-time *% (n)*	72% (84)	61% (86)	.06
Year of graduation, *M ± SD*	1986 ± 12	1989 ± 10	.02
Years worked in current area, *M ± SD*	14 ± 11	13 ± 10	.59
Smoking status			.02
Daily smoker *% (n)*	9% (10)	4% (6)	
Occasional smoker *% (n)*	3% (3)	7% (9)	
Former smoker *% (n)*	36% (42)	22% (30)	
Never smoker *% (n)*	53% (61)	67% (90)	
Stages of Change (for smokers)			.95
Pre-contemplating quitting % (n)	33% (4)	29% (4)	
Contemplating quitting % (n)	58% (7)	64% (9)	
Currently in process of quitting % (n)	8% (1)	7% (1)	
Time spent intervening with tobacco % (n)			.12
0-3 minutes	35% (39)	42% (58)	
3-10 minutes	35% (39)	21% (29)	
10+ minutes	4% (4)	4% (5)	
Does not counsel	27% (30)	33% (45)	
Provides tobacco cessation talks ^a ^M ± SD	1.2 ± 0.6	1.1 ± 0.4	.15
Believe brief advice to stop smoking is effective^b^	2.4 ± 0.9	2.4 ± 1.0	.58
Attitude about intervening ^b, c ^M ± SD	3.2 ± 0.5	3.1 ± 0.5	.18
Confidence with intervening ^d ^M ± SD*	2.6 ± 0.7	2.3 ± 0.6	< .01
Tobacco counselling perceived as part of role			.01
Very much	44%	28%	
Somewhat	53%	63%	
Not at all	3%	9%	

Note. M represents the arithmetic mean and ± SD represents 1 standard deviation. There were less than 6 missing per group for all analyses except year of graduation--rural was missing 7 and urban was missing 14. The p value was set at α = .01 using a Bonferroni adjustment for multiple comparisons for demographics and beliefs/confidence, and time spent.

^a ^Scale was 1 = never, 2 = seldom, 3 = occasionally, and 4 = frequently.

^b ^Scored on a 4-point scale from 1 (strongly disagree) to 4 (strongly agree).

^c ^The mean represents the average of the 4 attitude items.

^d ^Scored on a 4-point scale from 1 (not confident) to 4 (very confident). The mean represents the average of the 8 confidence items.

### Systems-level clinical practice guidelines

There were significant rural-urban differences in the proportion of nurses reporting having systems-level cessation supports, with less than half of the urban nurses reporting any systems. Specifically, significantly more rural nurses reported having a tobacco documentation system, cessation resources in the hospital (self-help materials, posters, and quit line contact information), and policies to identify tobacco use and document cessation counselling (Table 2). There were no urban-rural differences on having received cessation training (11% overall had training), having community-based cessation resources in the hospital (27%), or policies to provide cessation counselling (36%).

**Table 2:** Systems to support smoking cessation interventions

Systems Recommendations	Rural (N = 116) % yes (n/N)	Urban (N = 141) % yes (n/N)	p
Tobacco documentation system^a^	58% (65/112)	33% (46/139)	< .01
Cessation resources in hospital^a^			
Pamphlets/self-help materials	72% (81/112)	46% (63/138)	< .01
Posters	56% (61/109)	36% (50/138)	< .01
Quit line contact information	38% (42/110)	21% (28/136)	< .01
Community-based information	31% (34/111)	24% (32/136)	.21
Received cessation training^b^	16% (18/115)	7% (10/140)	.03
Policy to identify tobacco use^a^	60% (68/114)	44% (61/139)	< .01
Policy to provide counselling^a^	33% (36/108)	39% (52/133)	.35
Policy to document counselling^a^	29% (31/107)	14% (19/132)	< .01

Note: The p value was set at α = .01 using a Bonferroni adjustment for multiple comparisons.

^a ^Answer options yes, no, and unsure.

^b ^Answer options yes and no.

### Training

There were no rural-urban differences for the preferred training format or content. Format preferences included anything brief--in-service trainings during meetings (40%), self-study (38%), and 1-hour or half-day workshops (34% each); few wanted full-day workshops (20%). The preferred training topics included assessing readiness to quit (73%), counselling (62%), and how to find/recommend post-discharge services (62%). Preferences for specific counselling topics included: providing advice (57%), choosing self-help materials (58%), providing social support (57%), and helping patients recruit social support (57%). There was little interest in asking patients about tobacco use (20%), likely because most nurses reported asking as part of their practice. There were two significant rural-urban differences: more rural nurses wanted to know how to counsel patients who continued to smoke (47% vs. 29%, p ≤ 0.01) and how to organize files for patient follow-up (31% vs. 17%, p ≤ 0.01).

### Interventions in practice

**Figure 1:**
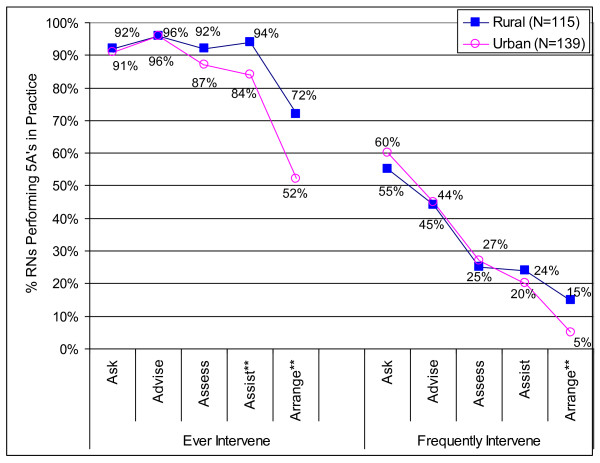
**Adherence to the 5A Protocol (N = 254)**. Note: The p value was set at α = .01 using a Bonferroni adjustment for multiple comparisons. Respondents were coded as frequently intervening if they frequently performed any of the activities within a given overall section of the 5A protocol (ask, advise, assess, assist, and arrange), and were coded as ever intervening if they ever performed any of the activities within a given section of the 5A protocol, even if seldom. ** p ≤ 0.01.

The 5A section of the questionnaire was completely blank for three respondents, all of whom were new hires; they were excluded from the analyses. The findings showed that almost all nurses had *asked, advised, assessed*, and *assisted *in the previous year, even if only seldom, but less than half did so frequently (Figure 1). There was no difference between rural and urban nurses in initiating interventions *(asking, advising*, and *assessing*), but rural nurses were significantly more likely to *assist *and *arrange *follow-up.

In relation to specific activities, the most frequently performed were those that could be done in a short period of time--asking about tobacco, advising to quit, explaining the harmful effects, encouraging/motivating patients to quit, and suggesting NRT, actions to cut down, and smoking alternatives (Table 3). The least frequently performed activities were those that would potentially take more time or require counselling skills (negotiating a quit date, teaching coping skills, and discussing patients' tobacco use with family members), and those that involved pharmacotherapy or referral to other resources or providers. Rural nurses intervened significantly more often than urban nurses on explaining how tobacco contributed to patients' illnesses (*advise*), *assisting *patients to quit (on all but two activities in Table 3), and *arranging *referrals.

**Table 3:** Details of the types of tobacco intervention activities performed in daily nursing practice (N = 254)

	Rural (N = 115) **M (SD)**^**a**^	Urban (N = 139) **M (SD)**^**a**^	p value
***Ask***			
Assess tobacco use status and history	3.2 (1.0)	3.3 (1.0)	.51
***Advise *(overall mean score)**	**2.8 (0.8)**	**2.8 (0.7)**	**.66**
Advise to quit using tobacco	2.8 (1.0)	2.9 (1.0)	.83
Explain tobacco use effects	2.8 (0.9)	2.9 (0.8)	.41
Explain second-hand smoke effects	2.7 (1.0)	2.8 (0.9)	.66
Explain how tobacco contributed to illness	2.8 (0.9)	2.5 (1.0)	.01
***Assess *(overall mean score)**	**2.2 (0.8)**	**2.1 (0.8)**	**.19**
Encourage patients to quitting again	2.5 (1.0)	2.5 (1.1)	.66
Motivate patients to quit	2.6 (1.0)	2.4 (1.1)	.16
Help set a quit-date	1.6 (0.8)	1.4 (0.6)	.10
***Assist *(overall mean score)**	**2.1 (0.7)**	**1.7 (0.6)**	**< .01**
Suggest actions to quit or cut down	2.5 (0.9)	2.2 (1.0)	.01
Use written materials to help patients	2.4 (1.0)	1.6 (0.9)	< .01
Recommend alternatives for tobacco	2.3 (1.0)	2.1 (1.0)	.14
Recommend or suggest NRT	2.2 (1.0)	1.9 (1.0)	.01
Teach coping skills to prevent relapse	2.0 (0.9)	1.7 (0.9)	.01
Instruct on the use of pharmacotherapy	2.0 (0.9)	1.6 (0.9)	< .01
Recommend or suggest bupropion	1.8 (1.0)	1.4 (0.7)	< .01
Discuss patients' tobacco use with family	1.5 (0.8)	1.4 (0.6)	.04
***Arrange *(overall mean score)**	**2.1 (0.9)**	**1.6 (0.7)**	**< .01**
Refer to cessation resources	2.1 (1.0)	1.7 (0.9)	< .01
Refer to other healthcare professionals	2.0 (1.0)	1.5 (0.8)	< .01

Note: Scale was 1 = never, 2 = seldom, 3 = occasionally, and 4 = frequently. Using a Bonferroni adjustment for each family of comparisons for the 5A's, resulted in the p value set at: "ask" α = .05, "advise" α = .01, "assess" α = .02, "assist" α = .01, and "arrange" α = .03.

^a ^M (SD) represents the arithmetic mean plus or minus one SD.

### Facilitating factors

**Figure 2:**
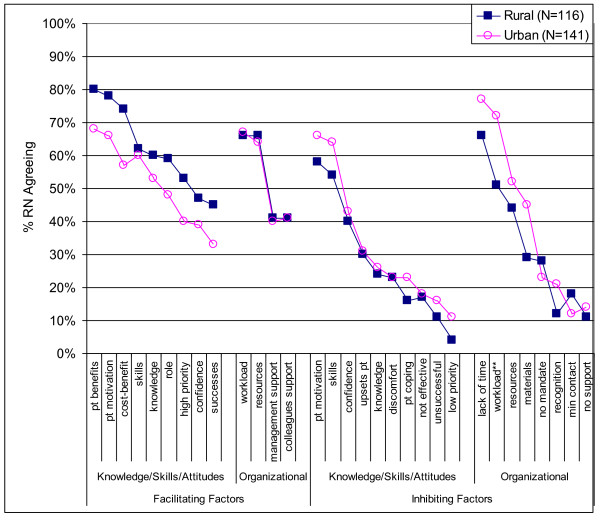
**Factors that facilitate and inhibit nurses from intervening with tobacco cessation interventions (N = 257)**. Note: "pt" represents 'patient'. Factors that facilitate and inhibit nurses from intervening were measured as check-all-that-apply type questions. The p value was set at α = .004 for facilitating factors and α = .003 for inhibiting factors using a Bonferroni adjustment for multiple comparisons. ** p = .001.

Patient-related factors (knowledge of patient benefits of quitting and patient motivation) encouraged most nurses to intervene (Figure 2). There were no rural-urban differences. Open-ended comments in relation to facilitating factors included personal experience with the harmful effects of tobacco, patient interest, patient requests for information and help, and systems to support interventions. The systems comments included in-service education, understanding adult education principles, information on pharmacotherapy, a standardized approach and reminders on the unit with phone numbers for referral, more time to be with the patient to provide the support, and being part of a supportive team for tobacco cessation rather than the primary counsellor.

### Inhibiting factors

There were only four factors that at least 50% of respondents agreed discouraged them from intervening--lack of patient motivation, lack of counselling skills, heavy workload, and lack of time (Figure 2). The only rural-urban difference was more urban nurses were discouraged from intervening due to workloads (p = .001). Open-ended comments in relation to inhibiting factors included mention that the unit they worked on was either not relevant for intervening (neonatal and paediatrics) or inappropriate and limited in terms of time (ICU and emergency). Other comments included not being interested in intervening and not believing that intervening was part of their role because "*nurses are not counsellors or doctors and cannot recommend, prescribe, or suggest alternatives such as pharmacotherapy*". Some nurses noted that they did not intervene because it was an infringement on patients' right to smoke (e.g., "*acutely ill patients are not interested in hearing why they shouldn't smoke"; "forcing patients to quit smoking is judging them which is NOT our role"; "smoking is all that's left for palliative patients and if they wish to smoke the only harm is second hand smoke exposure to others"*). There were also comments that a "*majority*" of nurses and management smoke which discourages intervening with patients. Others mentioned that it is hard to advocate for cessation when people are just outside the hospital entrance smoking and when there are no resources, either written materials or clinics, to refer patients to for quitting smoking.

## Discussion

The picture that emerged from this pre-program evaluation was encouraging, at least from the perspective of those nurses who took the opportunity to share their views. Both rural and urban nurses held relatively positive attitudes toward advising patients to quit, and despite a general lack of intervention training and little agreement that brief advice to quit is effective (as has been found in other studies [12]), the majority spent at least some time intervening. The most frequently performed activities tended to take the least amount of time, while the more complex activities (e.g., teaching coping skills and pharmacotherapy education) were seldom performed. Patient-related factors (quitting benefits and motivation) encouraged nurses to intervene and workloads and lack of time discouraged them, the latter of which is consistent with the literature [15]. Unexpected were the significant differences between rural and urban nurses. Rural nurses were more likely to perceive intervening as part of their role, had higher confidence for intervening, more frequently assisted patients with quitting and arranged follow-up, reported having more systems in place to support cessation, and were less likely to report workload as being a barrier to intervening.

Although the response rate was relatively low, interpretation of the findings was enhanced by using previously published survey items with good internal reliability and comparing the outcomes to the study on which the current study was based and which had a high response rate [13]--the outcomes of the 5A protocol steps, especially for rural nurses, were remarkably similar between the two studies. Compared to other studies, the levels of "ever intervening" with tobacco in the current study were substantially higher but "frequently intervening" was lower [11,12]. However, it is difficult to compare with other studies due to inconsistencies of measurement scaling and reporting across studies [21]. Some researchers treat interval scales as categorical and report proportions by scale option for each survey item (e.g., % reporting frequent, occasional, seldom, and never) without presenting an overall percentage for each of the global 5A steps [14], while others report the proportion who have ever done a given activity, without reference to frequency [22]. Some studies use "unbalanced" rating scales (i.e., an unequal number of favourable responses at one end of the scale, such as "almost always" and "frequently"), which tends to result in more positive responses [23]; these studies then dichotomize the reporting of "frequently" intervening using the top two positive responses [11,12], whereas "frequently" in the current study represented only the top anchor choice. These methodological inconsistencies suggest the need for more research in this area to develop standardized measures and methods of reporting.

The rural-urban differences were important findings, and given there were no differences in cessation training, might be reasonably attributed, at least in part, to the uniqueness of the rural context [24]. Positive role perception for tobacco interventions, for example, might derive from the fact that rural nurses often have an expanded role of practice [24] and from our experience, they take on a number of different roles within a single nursing position such as staff educator, manager of chronic diseases, and telehealth coordinator, and within any one of their roles, there might be a clear indication for tobacco cessation treatment. Increased confidence might be due to the need for a wide range of knowledge and skills in rural nursing, which is influenced by the community demographics and the need to work with different populations in different areas of the hospital [24], often all in the same day, which in turn, represents a level of cross-training not common in larger centres. The higher rural smoking rates could have influenced intervention frequency as nurses are faced with various consequences of smoking across the units they work on, and they tend to be responsive to their community's needs because, as other studies have shown, "we see those people in our churches and in our grocery stores...in the end, we are the ones who see these people outside of our work life too." (pg 22) [24]. In the USA, nurses in states with higher smoking rates vs. lower rate also intervene more often [11].

In relation to the systems differences between urban and rural nurses, rural nurses might be more aware of systems such as patient materials due to the physical environment layout. Nursing stations in the participating rural hospitals are commonly shared among many units and serve as central storage areas for staff and patient education materials. Most of the rural hospitals also have patient self-help materials on wall-racks located in the main foyer, which serves as the reception/waiting area, and through which staff pass on their way to/from work. Relative to documenting tobacco use/counselling, the rural hospitals used paper charting so every form is physically in the nurses' hands and easy to see. In contrast, each form in the urban hospital's electronic charting system has a separate tab that needs to be pulled down, and the cessation counselling tab in the urban hospital was optional, relatively new, and not well-promoted.

Overall, the findings provide insight into what is likely realistic in the context of staff nurses' daily practice. Although training would be expected to increase the frequency of intervening [8], the activities (those that could be done in a short period of time) and time (< 10 minutes) that the nurses reported in relation to intervening, and the most frequently noted barriers to intervening (time and workload) indicate that what nurses are willing and able to do maps onto the brief versus intensive interventions recommended by the Guidelines [8]. In turn, brief interventions seem reasonable given the context of today's faster paced healthcare environment in which patient contact hours have been reduced, units are downsizing, and patient acuity and turnover have increased [25]. Even brief interventions, however, are not necessarily easy to maintain--training must be ongoing and can be complex in larger hospitals; there is often staff resistance and a lack of accountability when the responsibility is diffused over a large number of staff; it is difficult to measure outcomes because to do so requires standardized charting and doing chart reviews; and brief interventions require some form of central organization and funds from the operating budget, even if only for training [26]. The activities that the nurses reported doing less frequently are more in line with more intensive interventions that require more time, more training, and often a dedicated staff position.

### Implications for practice

It was encouraging that the majority of nurses would likely be agreeable to providing cessation interventions as part of standard practice, and that they would not have to be convinced about the importance and desirability of intervening as much as it would be important to develop an intervention that would fit with the realities of their busy practice. Based on this needs assessment, we developed a brief intervention consistent with the Guidelines [4,8] with the *assist *and *arrange *steps consisting primarily of distributing printed materials and avoiding the more complex types of counselling. For training, we developed a 30-minute in-service being mindful of nurses' heavy workloads and preferences for training, and provided information where nurses could engage in more in-depth training if interested [4]. To highlight the patient benefits of quitting, which was the primary encouraging factor for intervening, we included smoking rates for each community in the training, as well as additional risks to hospitalized smokers and immediate benefits of quitting. We also included information from the Guidelines [4,8] to increase nurses' knowledge of what was being recommended and to highlight the effectiveness of brief interventions. To enhance nurses' confidence to provide brief interventions, we demonstrated how to perform the 5A's in 1-3 minutes.

### Limitations

One limitation of this study is that the data were self-reported and not validated by medical charts. Other studies suggest a tendency to over-report (vs. under-report) desirable cessation-related activities [27]. Another limitation is the relatively low response rate. Although the response rate was within the expected range [28] and the outcomes were similar to the study [13] on which the current study was based, the nurses who responded to the survey might not be representative of the target population and thus the outcomes are not necessarily generalizable to all acute-care nurses working in the region or in other areas of the province or country. Obtaining a high survey response rate, especially in the fast-paced environment of acute care, can be difficult to achieve and is one of the disadvantages of survey research [27].

## Conclusions

The findings showed nurses' willingness to engage in tobacco interventions and that the majority were doing something although the frequency of intervening was suboptimal. The time spent and types of activities nurses engaged in were consistent with the recommended minimum [4,8] and could be interpreted as realistic for the acute care environment. We were able to recognize strengths and gaps in practice from nurses' responses, and build on those in the development of a brief intervention and training thereby highlighting the importance of pre-program evaluation. The rural-urban differences add to the literature and suggest a need for researchers and nurse leaders to look more closely at the context of rural nurses to capture the distinctiveness and to highlight the strengths, a "rural lens", as it has been called, which in turn, might be helpful in informing approaches in urban settings [24].

## Competing interests

The authors declare that they have no competing interests.

## Authors' contributions

PMS and SMS were responsible for the study design and implementation strategy as well as all aspects of data collection. PMS was responsible for the data analysis and interpretation, initial write-up, and revisions; SMS contributed to final iterations of the initial paper and revisions; MMS contributed to data interpretation and final iterations of the paper; all authors reviewed and approved the final manuscript.

## Pre-publication history

The pre-publication history for this paper can be accessed here:

http://www.biomedcentral.com/1472-6955/11/6/prepub
